# Human/eukaryotic ribosomal protein L14 (RPL14/eL14) overexpression represses proliferation, migration, invasion and EMT process in nasopharyngeal carcinoma

**DOI:** 10.1080/21655979.2021.1932225

**Published:** 2021-05-30

**Authors:** Zunni Zhang, Yalong Zhang, Yuling Qiu, Wuning Mo, Zheng Yang

**Affiliations:** a Department of Clinical Laboratory, First Affiliated Hospital of Guangxi Medical University, Nanning, Guangxi Zhuang Autonomous Region, China; b Department of Ultrasonic Medicine, First Affiliated Hospital of Guangxi Medical University, Nanning, Guangxi, China

**Keywords:** Nasopharyngeal carcinoma, RPL14(eL14), EMT process, ribosomal protein, progression

## Abstract

Although human/eukaryotic ribosomal protein L14 (RPL14/eL14) is known to be associated with a variety of cancers, its role in nasopharyngeal carcinoma (NPC) remains unclear. The aim of this study was to explore the impact of RPL14(eL14) in NPC. The results of quantitative real-time polymerase chain reaction (qRT-PCR), western blot, and immunohistochemical staining revealed that the expression of RPL14(eL14) significantly reduced in NPC tissues and cells. Furthermore, the protein expression of RPL14(eL14) was linked to NPC-related clinical pathological features, including the T and N classification of Tumor Node Metastasis (TNM) staging (all p < 0.05). Cell counting kit-8 (CCK-8) assay and colony formation assay revealed that RPL14(eL14) overexpression repressed NPC cell proliferation. In cell cycle assay, RPL14(eL14) overexpression significantly blocked NPC cells in S phase. Overexpression of RPL14(eL14) repressed cell migration and invasion in NPC as shown by transwell assay and cell scratch healing assay. In addition, RPL14(eL14) was closely correlated with the expression of epithelial–mesenchymal transition (EMT) biomarkers, including E-cadherin, N-cadherin, and vimentin as detected by western blot. In conclusion, our results revealed that RPL14(eL14) may be considered as an antioncogene in NPC, which greatly suppresses cancer progression.

## Graphical Abstract

**Figure:**
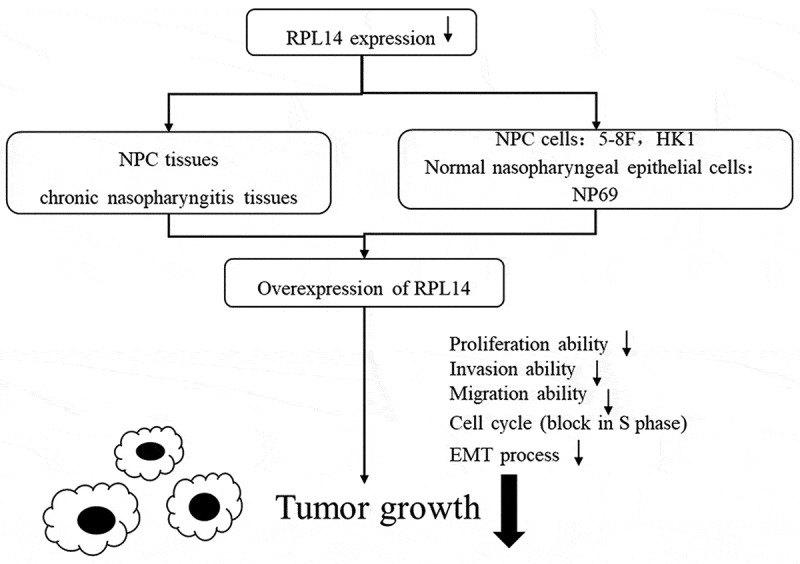

## 1. Introduction

Nasopharyngeal carcinoma (NPC) is one of the most common head and neck tumors, which often occurs in the epithelial lining of the nasopharynx [1,2]. There are differences in the geographic and ethnic distribution of nasopharyngeal cancer, with NPC common among populations in South China, North Africa, and Southeast Asia [3,4]. At present, the main methods for NPC treatment are chemotherapy, and radiotherapy [4]. With the advances in radiotherapy and chemotherapy technology, the 5-year survival rate in NPC patients has increased from 25% to 40% to about 70%, which points to a marked improvement in the overall prognosis of NPC [5]. The increase in NPC-related mortality is due to local recurrence and metastasis after treatment [6]. The occurrence and development of NPC is a complex process. Previous studies showed that environmental factors, genetic factors, and Epstein–Barr virus infection were the main causes of NPC [7,8]. The progression of NPC is associated with genetic changes in specific chromosomal regions and genes and includes proto-oncogene activation, tumor suppressor gene inactivation, single nucleotide polymorphisms (SNPs), and epigenetic changes [9]. Therefore, we believe that it is very important to further explore the mechanisms underlying metastasis and growth in NPC.

Human/eukaryotic ribosomal protein L14 (RPL14/eL14) is a member of the L14E ribosomal protein family. The protein consists of five introns and six exons and is located on chromosome 3p21.3. Previous research has linked RPL14(eL14) with esophagus squamous cell carcinomas (ESCC) [10], lung and oral cancers [11], cervical cancer [12], colorectal cancer [13] and glioblastoma multiforme [14]. Sun et al. reported that RPL14(eL14) can rescue the progression role of miR-129-5p in cervical cancer cells [15]. In triple-negative breast cancer patients, the reduced expression of RPL14(eL14) was associated with lower survival rates [16]. Thus, literatures suggest that RPL14(eL14) is related to tumorigenesis. At present, there are rarely any data concerning the role of RPL14(eL14) in NPC.

The epithelial–mesenchymal transition (EMT) refers to the biological process of epithelial cells transforming into mesenchymal cells through specific procedures. The EMT is an important mechanism in tumor cell migration, invasion, and involves downregulation of E-cadherin expression and increased expression of vimentin and N-cadherin [17]. In 2012, Lo et al. proposed that deletion of chromosome 3p and 9p regions was a prerequisite for normal nasopharyngeal epithelial transformation [18]. A number of studies reported that various epigenetic and transcriptional factors regulated the EMT process [19,20]. However, the role of RPL14(eL14) in EMT is still unknown.

Although RPL14(eL14) has been found to be associated with a variety of cancers, the role of RPL14(eL14) in NPC remains unclear. Therefore, the aim of this study was to examine the RPL14(eL14) expression in NPC and then examined the role of RPL14(eL14) in NPC cell proliferation, cell cycle, cell migration, invasion, and the EMT process.

Our data strongly suggested that RPL14(eL14) contributes to the NPC growth and metastasis. Thus, we suggested that RPL14(eL14) may be considered as an antioncogene in NPC.

## 2. Materials and methods

### 2.1. Clinical samples

From July 2017 to August 2018, we collected 16 NPC tissues (male/female = 13:3, age: 51 ± 11.5) and 16 chronic nasopharyngitis tissues (male/female = 10:6, age: 48 ± 13.1) from the First Affiliated Hospital of Guangxi Medical University, Nanning, China for quantitative real-time polymerase chain reaction (qRT-PCR) assay. At the same time, 32 chronic nasopharyngitis tissues and 86 NPC tissues were obtained and used for immunohistochemistry assay. MRI and serum Epstein-Barr virus detections were performed on the patients suspected of NPC. If NPC was indeed suspected, nasopharyngeal biopsy could be performed for pathological diagnosis. All the patients had been recently diagnosed with NPC. The diagnosis had been confirmed pathologically. They had no other tumors and had not undergone chemotherapy or radiotherapy prior to sample collection. Two senior pathologists performed the tumor staging, with staging adhering to the Tumor Node Metastasis (TNM) staging method of the International Union Against Cancer (eighth edition). All surgically isolated fresh tissues were placed in labeled 1.5 ml enzyme-free EP tubes and stored in liquid nitrogen before RNA extraction and tissue section.

All the participants signed an informed consent form. The study was approved by the ethics committee of the First Affiliated Hospital of Guangxi Medical University, approval number: 2021(KY-E-092).

### 2.2. Cell culture

Cell lines (NP69, 5–8 F, and HK1) were obtained from the Otolaryngology Laboratory of Guangxi Medical University, Nanning, China. The cells were cultured in complete cell culture medium, which was mixed with 89% RPMI – 1640 medium (Gibco, USA), 10% fetal bovine serum (FBS; Gibco, USA), and 1% penicillin-streptomycin mixture (Solarbio, China) at 37°C with 5% CO^2^.

### 2.3. Immune-histochemical staining

After rehydration, the tissue specimens were incubated in citrate buffer (pH = 6.0) at high pressure for 8 minutes, followed by immersion in 3% hydrogen peroxide at room temperature for 15 minutes. After incubation of the tissues with anti-RPL14(eL14) overnight, the tissue specimens were incubated with biotin-labeled goat anti-rabbit secondary antibody at room temperature for 30 minutes, followed by DAB color development for 5 min and hematoxylin staining for 1 minute. The immunohistochemical staining result was judged by the cell staining intensity and the percentage of positive stained cells [21].

### 2.4. Stable construction of RPL14(eL14) overexpression cell lines

Control vector (LV-pGV) and lentiviral expression clones for RPL14(eL14) (LV-RPL14) were purchased from Genechem, Shanghai, China. Lentiviral infection solution was added to the cells according to the manufacturer’s instructions, and the cells were cultured for 48 hours. Subsequently, stably transfected cells with puromycin for 10 days were selected. Finally, the successful construction of RPL14(eL14) overexpression cell lines was verified by qRT-PCR and western blot assays.

### 2.5. Quantitative real-time PCR (qRT-PCR)

In brief, total RNA was extracted from the tissues and cells by Trizol reagent (TaKaRa, Japan). Then, cDNA was synthesized using All-In-One 5X RT MasterMix (Applied Biological Materials Inc., USA). To detect RPL14(eL14) expression, a qRT-PCR assay was performed using an EvaGreen 2X qPCR MasterMix kit (Applied Biological Materials Inc, USA). Primers were synthesized by Sangon Biotech (Shanghai, China). The primer sequences for qRT-PCR were as follows: GAPDH-forward: 5ʹ- TGGGTGTGAACCATGAGAAG-3ʹ, GAPDH- reverse: 5-’ GTGTCGCTGTTGAAGTCAGA-3ʹ. RPL14(eL14)-forward: 5-’TTAAGAAGCTTCAAAAGGCAGC-3ʹ, RPL14(eL14)- reverse: 5-’TTTTGACCCTTCTGAGCTTTTG-3ʹ.

### 2.6. Western blot

For protein extraction, a mixture of 90% RIPA lysis buffer and 10% protease inhibitor (PMSF; Solarbio, China) was used. After SDS-PAGE gel electrophoresis at 120 V for 1 hour and transfer of the proteins to a PVDF membrane at 150 mA for 90 minutes, the PVDF membrane was incubated with corresponding anti-RPL14(eL14), anti-GAPDH, anti-E-cadherin, anti-N-cadherin, and anti-vimentin (Abcam, UK) overnight, then incubated in fluorescent antibody (Thermo Fisher, USA) for 1 hour. GAPDH served as a control. The results were quantitative by image J software [22].

### 2.7. Cell counting kit-8 (CCK-8) assay

The cells were digested by trypsin, and the cell resuspension solution (5–8 F:1 × 10^3^ cells/well, HK1: 2 × 10^3^ cells/well) was plated in 96-well plates. The absorbance of CCK-8 (OD 450 nm) 0, 24, 48, and 72 hours after cell attachment was then measured. The absorbance at OD 450 nm reflected the cell proliferation rate [23].

### 2.8. Colony formation assay

The cells (5–8 F: 500 cells/well, HK1: 800 cells/well) were plated in six-well plates and cultured in medium for 14 days. Subsequently, the cells were fixed in 4% paraformaldehyde for 20 minutes and then stained with 1% crystal violet for 20 minutes. More than 50 cells counted as one clone. The cell clone formation rate = the number of clones/number of inoculated cells × 100% [22].

### 2.9. Cell cycle assay

We used 0.25% no EDTA trypsin to digest the cells, followed by resuspension with 70% cold ethyl alcohol at 4°C for 24 hours. Then, 100 µl of 10 mg/ml RNase were added to the cells, and the cells were incubated at 37°C for 30 minutes, followed by cell staining with propidium iodide at 4°C for 30 minutes. The cell cycle was analyzed by flow cytometry (BD Biosciences, USA) and visualized by Modfit software (version 3.1).

### 2.10. Transwell migration and invasion assay

In the transwell invasion assay, the cells (2 × 10^2^) were seeded with FBS-free medium into the upper chamber of an 8-μm pore membrane containing Matrigel (transwell migration had no Matrigel). In the lower chamber, 10% FBS medium was added. After culturing for 48 hours, the membrane was fixed with 4% paraformaldehyde for 20 minutes, and the cells were then stained with 1% crystal violet for 20 minutes. The number of cells that passed through the membrane indicated the migration and invasion ability of tumor cells [22].

### 2.11. Cell scratch healing assay

In the six-well plates overgrown with cells, a sterile 200 μl pipette tip was used to draw a blank area on the confluent monolayer of the cells. The wells were then carefully washed three times with a PBS buffer and photographed using an inverted microscope (Olympus, Japan) to record the distance of the scratched area and marked them as D0. The cells were then incubated at 37°C with 5% CO^2^ with FBS-free RPMI-1640 medium for 48 hours and marked as D48. The cell scratch healing rate reflected the cell migration ability. The cell scratch healing rate = (D0 – D48)/D0 × 100%.

### 2.12. Statistical analysis

All data were analyzed using SPSS software, version 24.0. The difference between the two groups was analyzed using the Student’s t-test. The data are presented as mean ± standard deviation. The Mann–Whitney U-test was also conducted, with the data presented as median and interquartile range. The relation between RPL14(eL14) expression and clinical characteristics was analyzed using the χ2 test. Each assay was repeated at least three times. A p value of <0.05 (two-tailed) was considered a statistically significant difference.

## 3. Results

RPL14(eL14) was down-regulated and may serve as an antioncogene in NPC. Multiple experiments revealed that RPL14(eL14) inhibited NPC cell proliferation, invasion, migration and EMT processes. Moreover, RPL14(eL14) blot NPC cells in S phase. All of the results suggested that RPL14(eL14) may affect the occurrence and development of NPC.

### 3.1. The down-regulation of RPL14(eL14) in NPC tissues and cells

**Figure 1.**
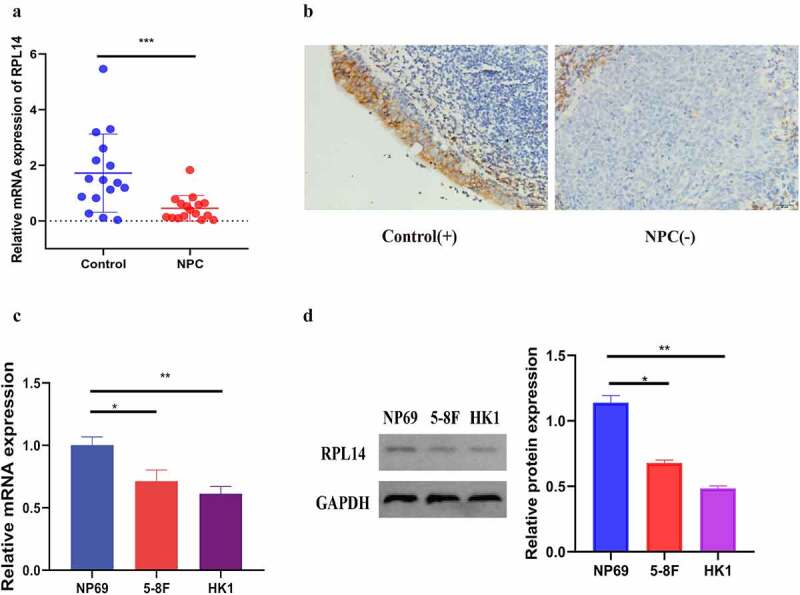
RPL14(eL14) is down-expression in NPC. The relative mRNA expression of RPL14(eL14) in NPC and chronic nasopharyngitis tissues (a). The immumohistochemical staining of RPL14(eL14) in NPC and chronic nasopharyngitis tissues (b). RPL14(eL14) is down-expression in normal nasopharyngeal epithelial cell line (NP69) when compared with NPC cell lines (5–8 F and HK1) based on qRT-RCR and western blot (c,d). *p < 0.05, **p < 0.01 and ***p < 0.001

Figure 1(a) shows the relative mRNA expression levels of RPL14(eL14) in the tissues of the chronic nasopharyngitis cases (n = 16) and NPC (n = 16) cases. The mRNA expression in the NPC tissues was significantly low as compared with that in the control tissues (p < 0.001).

Immunohistochemical staining was utilized to test the protein expression of RPL14(eL14) in 32 chronic nasopharyngitis tissues and 86 NPC tissues. Consistent with the mRNA expression, the protein expression of RPL14(eL14) was weakly detected in the NPC tissues, with a 31.395% positive rate. In the 32 chronic nasopharyngitis tissues, 53.125% exhibited high expression of RPL14(eL14) (Figure 1(b), Table 1, ‘-’ represents negative, ‘+’ represents positive). In addition, RPL14(eL14) expression showed a significant correlation with the NPC T classification (p = 0.005) and N classification (p = 0.003) (Table 2).

**Table 1.** The expression of RPL14(eL14) in normal nasopharyngeal epithelium and nasopharyngeal carcinoma tissues

Group	N	RPL14(eL14) expression		Positive rate (%)	p-value
		Positive(+)	Negative (-)		
Control	32	17	15	53.125%	0.03
NPC	86	27	59	31.395%

**Table 2.** The correlation between the expression of RPL14(eL14) and clinical characteristic in NPC

Variables	N	RPL14(eL14) expression		P-value
		Low (N,%)	High (N,%)	
Sex				
Male	24	16(66.67)	8(33.33)	0.81
Female	62	43(69.35)	19(30.65)	
Age				
<50	54	40(74.07)	14(25.93)	0.156
≥50	32	19(59.38)	13(40.63)	
T classification				
T1-T2	19	8(42.11)	11(57.89)	0.005
T3-T4	67	51(76.12)	16(23.88)	
N classification				
N0-N1	26	12(46.15)	14(53.85)	0.003
N2-N3	60	47(78.33)	13(21.67)	
M classification				
M0	73	50(68.49)	23(31.51)	0.958
M1	13	9(69.23)	4(30.77)	
Tumor stage				
I–II	8	6(75)	2(25)	0.993
III–IV	78	53(67.95)	25(32.05)	
lymphatic metastasis				
Yes	80	56(70)	24(30)	0.574
No	6	3(50)	3(50)	
Tumor histology				
differentiated carcinoma	10	5(50)	5(50)	0.324
undifferentiated carcinoma	76	54(71.05)	22(28.95)	

Consistent with the results from this study on NPC tissues, RPL14(eL14) mRNA and protein expression levels in the NPC cells (5–8 F and HK1) were significantly reduced when compared with the normal nasopharyngeal epithelial cells (NP69) (Figure 1(c,d)). Together, the results suggested that RPL14(eL14) may act as an antioncogene and inhibit tumor carcinogenesis in NPC.

### 3.2. RPL14(eL14) inhibited cell proliferation and cell cycle progression in NPC

**Figure 2.**
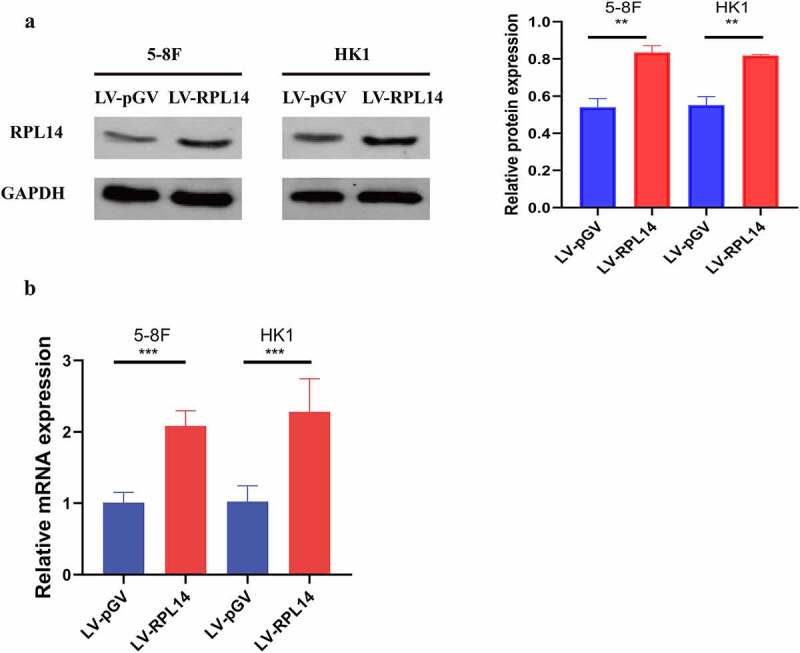
The protein and mRNA expression levels of RPL14(eL14) in 5–8 F and HK1 cells carrying RPL14(eL14) transgene (a,b). ** p<0.01, ***p < 0.001

To further explore the role of RPL14(eL14) in NPC, the overexpression of RPL14(eL14) in 5–8 F and HK1 cells were stably constructed by lentiviral. As shown by the results of both the western blot and qRT-PCR analyses, RPL14(eL14) expression was successfully overexpressed in 5–8 F and HK1 cells by a lentiviral (Figure 2(a,b)).

**Figure 3.**
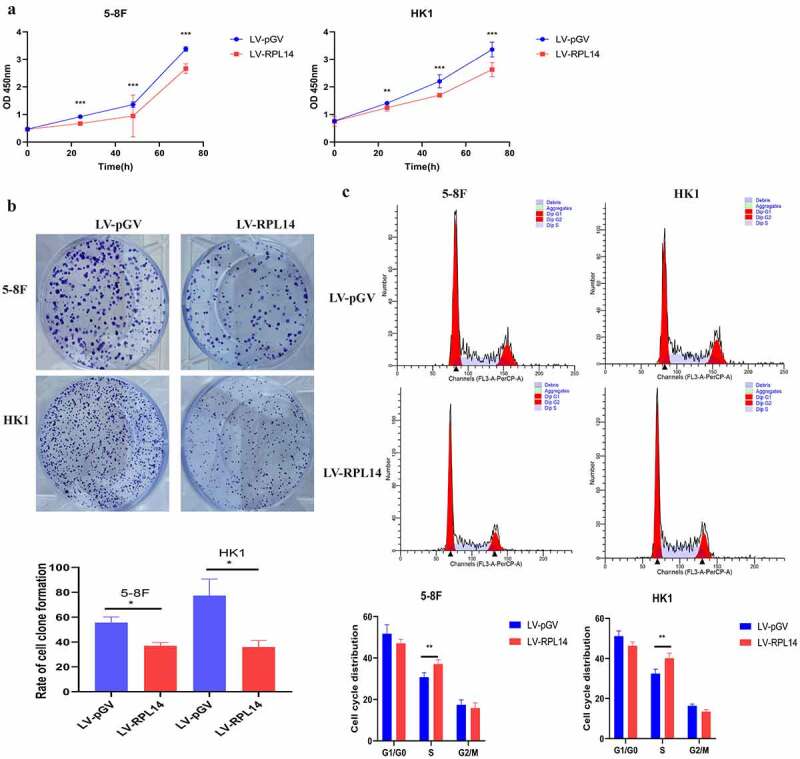
RPL14(eL14) inhibits proliferation and carcinogenesis in NPC. CCK-8 assay (a), colony formation assay (b). Cell cycle distribution (c). *p < 0.05, **p < 0.01 and ***p < 0.001

In the CCK-8 assay, proliferation of 5–8 F and HK1 with LV-RPL14 cells was significantly inhibited when compared with that of vector control groups after 24, 48, and 72 hours (all p < 0.05, Figure 3(a)). The colony formation assay revealed that the colony formation of 5–8 F and HK1 with LV-RPL14 cells were much less than in the vector control groups (all p < 0.05, Figure 3(b)).

Flow cytometry was utilized to analyze the role of RPL14(eL14) in cell cycle. In the 5–8 F cells overexpressing RPL14(eL14), the percentage of cells in the S phase was significantly higher than the percentage of cells in the S phase in the vector control group (37.10 ± 2.05% vs. 30.78 ± 2 .09%) (p < 0.05). However, there was no difference in the percentage of cells in the G1/G0 phase (15.8% vs. 17.43%) and G2/M phase (47.11% vs. 51.79%) (all p > 0.05). In terms of HK1 cells, the cells of S phase in LV-RPL14 were 40.16±2.51%, which was significantly higher than 32.45 ± 2.19% cells in LV-pGV (p = 0.004, Figure 3(c)).

### 3.3. RPL14(eL14) inhibited cell migration and invasion in NPC

**Figure 4.**
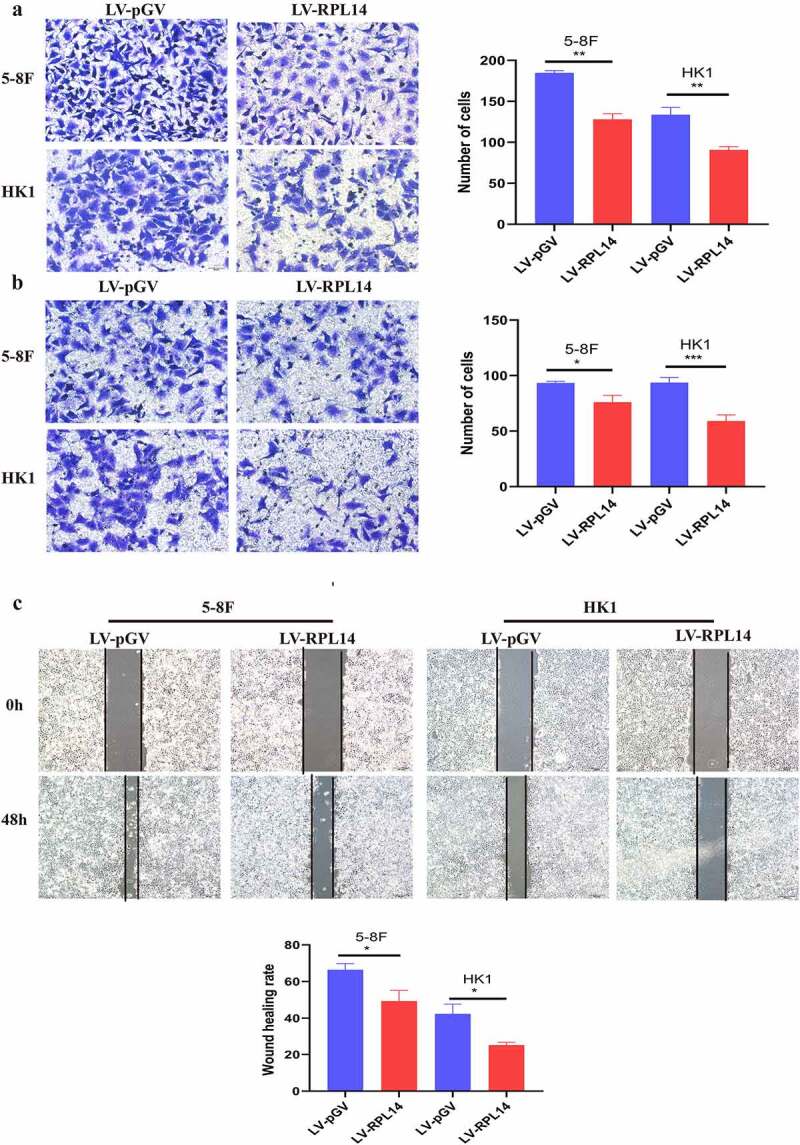
Overexpression of RPL14(eL14) inhibited tumor invasion and migration in NPC by transwell migration assay(a), transwell invasion assay(b), and scratch migration assay(c). *p < 0.05, **p < 0.01 and ***p < 0.001

The migration of NPC cells was first tested by transwell migration assay. As shown in Figure 4(a), overexpression of RPL14(eL14) notably repressed the migration of 5–8 F and HK1 cells (all p < 0.01). Consistent with the results of the transwell migration assay, the scratch healing migration assay showed that RPL14(eL14)-overexpressing 5–8 F and HK1 cells displayed dramatically lower mobility than the vector control groups (Figure 4(c), p < 0.05). As shown in Figure 4(b), overexpression of RPL14(eL14) significantly reduced the invasion of NPC cells (all p < 0.05).

Together, the results demonstrated that RPL14(eL14) overexpression repressed NPC cell migration and invasion.

### 3.4. RPL14(eL14) inhibited the EMT process in NPC

**Figure 5.**
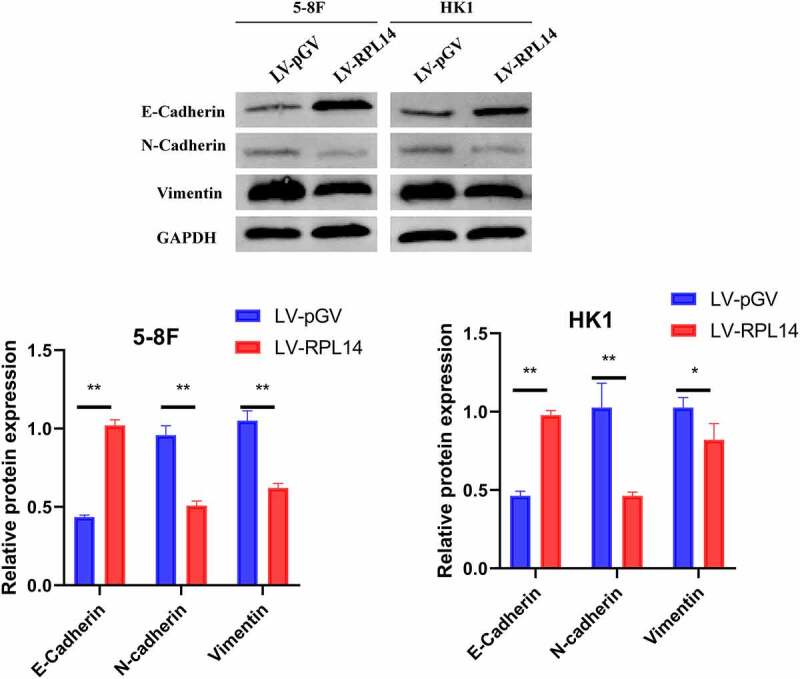
Overexpression of RPL14(eL14) on EMT biomarkers (E-cadherin, N-cadherin, and Vimentin) in 5–8 F and HK1 cells. *p < 0.05, **p < 0.01

The effect of RPL14(eL14) in 5–8 F and HK1 cells on the EMT process was determined by western blot. As displayed in Figure 5, in the groups of LV-RPL14, the expression levels of N-cadherin and vimentin were significantly reduced, whereas the expression levels of E-cadherin were obviously increased when compared with those in the control groups (LV-pGV). These results illustrated that RPL14(eL14) could inhibit the EMT process in NPC.

## 4. Discussion

It has been widely recognized that ribosomal proteins (RPs) were not only involved in the composition of ribosomes and biosynthesis of protein but also related to the occurrence and development of tumors [24]. For example, ribosomal S6 kinase 4 was reported to be a suppressor gene in a variety of cancers, including colorectal cancer [25], breast cancer [26], ovarian cancer [27] and acute myeloid leukemia [28]. Ribosomal protein L31 was found to regulate the proliferation of prostate cancer cells through the p53 pathway [29]. Xu et al. revealed that ribosomal protein L32 may be a new molecular therapy target in breast cancer [30]. Therefore, it may be of great value to explore the role of RPL14(eL14) in NPC.

We found that the loss of RPL14(eL14) was responsible for the development of NPC. Consistent with the findings of our research, a previous study revealed that RPL14(eL14) was down-expression and could serve as an early diagnostic biomarker for esophageal squamous cell carcinomas [10]. Shriver et al. reported that RPL14(eL14) was a cancer suppressor gene, which may play an important role in the pathogenesis of lung and oral cancers [11]. In a recent study, Yu et al. used bioinformatics analysis, identified RPL14(eL14) as a key gene in colorectal cancer [13]. However, for glioblastoma multiforme, RPL14(eL14) was upregulated and maybe accelerated the growth of tumors [14]. In addition, Feng et al. found that RPL14(eL14) promoted cancer invasion and migration in cervical cancer [12]. All these data not only indicated that the expression of RPL14(eL14) may have specific tissue heterogeneity [31,32] but also effectively confirm that RPL14(eL14) may serve as an important regulator in tumor progression.

Our proliferation assays revealed that RPL14(eL14) suppressed cell proliferation ability in NPC cells. To further analyze the possible roles of RPL14(eL14) in NPC, we performed cell cycle analysis. The results showed that overexpression of RPL14(eL14) arrested cell cycle in the S phase. Cell cycle arrest is a protective mechanism, which can be explained as when DNA is damaged, cell cycle monitoring points can be regulated to initiate cell cycle arrest for DNA repair. Cell cycle is mainly divided into G0/G1, S, and G2/M phases. Previous researches showed that S phase played an important role in the pathogenesis of a variety of cancers [33]. During the arrest of the S phase, some related signaling pathways were activated, leading to inhibition of cell proliferation [34]. Chen et al. revealed that the silencing of CBX4 suppressed cell proliferation by arresting cell cycle at S phase in cervical cancer [35]. Yang et al. showed that silencing of DOT1L inhibited cell proliferation and blocked cell cycle in the S phase in colorectal cancer [36].

Distant metastasis is the major reason for poor overall survival and treatment failure among NPC patients [37]. About two-fifths of loco-regional NPC patients progress to distant metastases after treatment [38]. It is difficult to detect subclinical micrometastases, due to there being no specific clinical manifestation of metastases in the clinical examinations and conventional health checkups. To the best of our knowledge, molecular biomarkers found to be associated with NPC metastasis, including lactate dehydrogenase and Epstein–Barr virus DNA [39]. However, the mechanism of NPC metastasis remains unclear [40]. As is well known, the EMT process involves transformation of epithelial cells into mesenchymal cells, which gives tumor cells the ability to metastasize [41]. E-cadherin is an adhesion marker of epithelial cells, and the decreased expression of E-cadherin contributes to a loss of intercellular connection [42]. The expression of vimentin and N-cadherin are thought to facilitate the progress of cell invasion, migration, and EMT process [43]. It was reported that several genes, including vascular endothelial growth factor [44] and forkhead box 1 [45], modulated NPC migration and invasion by regulating the EMT. In this study, overexpression of RPL14(eL14) notably repressed cell migration, invasion, and EMT process in NPC cells by reducing the expression of N-cadherin and vimentin and increasing the expression of E-cadherin. These results indicated that RPL14(eL14) may be involved in the modulation of NPC metastasis via the EMT process.

## 5. Conclusion

This research revealed that RPL14(eL14) both down-regulated in NPC tissues and cells as verified by a variety of experiments. The decrease of RPL14(eL14) was associated with few clinical features of NPC, including T and N classification. Moreover, multiple cell biological function experiments revealed that RPL14(eL14) may have inhibited the occurrence and development of NPC. However, these results need to be validated in future studies.

## Data Availability

The data supporting the results reported in the manuscript can be acquired by the corresponding author.

## Highlights

1. The role of RPL14(eL14) as an antioncogene in NPC.
2. RPL14(eL14) inhibited NPC cell proliferation.
3. RPL14(eL14) inhibited NPC cell migration, invasion and EMT process.
